# Predicting drug response of tumors from integrated genomic profiles by deep neural networks

**DOI:** 10.1186/s12920-018-0460-9

**Published:** 2019-01-31

**Authors:** Yu-Chiao Chiu, Hung-I Harry Chen, Tinghe Zhang, Songyao Zhang, Aparna Gorthi, Li-Ju Wang, Yufei Huang, Yidong Chen

**Affiliations:** 1 Greehey Children’s Cancer Research Institute, University of Texas Health Science Center at San Antonio, San Antonio, TX 78229 USA; 2 Department of Electrical and Computer Engineering, The University of Texas at San Antonio, San Antonio, TX 78249 USA; 3 Laboratory of Information Fusion Technology of Ministry of Education, School of Automation, Northwestern Polytechnical University, Xi’an, 710072 Shaanxi China; 4 Department of Epidemiology and Biostatistics, University of Texas Health Science Center at San Antonio, San Antonio, TX 78229 USA

**Keywords:** Deep neural networks, Pharmacogenomics, Drug response prediction, Cancer cell line encyclopedia, Genomics of Drug Sensitivity in Cancer, The Cancer Genome Atlas

## Abstract

**Background:**

The study of high-throughput genomic profiles from a pharmacogenomics viewpoint has provided unprecedented insights into the oncogenic features modulating drug response. A recent study screened for the response of a thousand human cancer cell lines to a wide collection of anti-cancer drugs and illuminated the link between cellular genotypes and vulnerability. However, due to essential differences between cell lines and tumors, to date the translation into predicting drug response in tumors remains challenging. Recently, advances in deep learning have revolutionized bioinformatics and introduced new techniques to the integration of genomic data. Its application on pharmacogenomics may fill the gap between genomics and drug response and improve the prediction of drug response in tumors.

**Results:**

We proposed a deep learning model to predict drug response (DeepDR) based on mutation and expression profiles of a cancer cell or a tumor. The model contains three deep neural networks (DNNs), i) a mutation encoder pre-trained using a large pan-cancer dataset (The Cancer Genome Atlas; TCGA) to abstract core representations of high-dimension mutation data, ii) a pre-trained expression encoder, and iii) a drug response predictor network integrating the first two subnetworks. Given a pair of mutation and expression profiles, the model predicts IC_50_ values of 265 drugs. We trained and tested the model on a dataset of 622 cancer cell lines and achieved an overall prediction performance of mean squared error at 1.96 (log-scale IC_50_ values). The performance was superior in prediction error or stability than two classical methods (linear regression and support vector machine) and four analog DNN models of DeepDR, including DNNs built without TCGA pre-training, partly replaced by principal components, and built on individual types of input data. We then applied the model to predict drug response of 9059 tumors of 33 cancer types. Using per-cancer and pan-cancer settings, the model predicted both known, including EGFR inhibitors in non-small cell lung cancer and tamoxifen in ER+ breast cancer, and novel drug targets, such as vinorelbine for *TTN*-mutated tumors. The comprehensive analysis further revealed the molecular mechanisms underlying the resistance to a chemotherapeutic drug docetaxel in a pan-cancer setting and the anti-cancer potential of a novel agent, CX-5461, in treating gliomas and hematopoietic malignancies.

**Conclusions:**

Here we present, as far as we know, the first DNN model to translate pharmacogenomics features identified from in vitro drug screening to predict the response of tumors. The results covered both well-studied and novel mechanisms of drug resistance and drug targets. Our model and findings improve the prediction of drug response and the identification of novel therapeutic options.

## Background

Due to tumor heterogeneity and intra-tumor sub-clones, an accurate prediction of drug response and an identification of novel anti-cancer drugs remain challenging tasks [1, 2]. Pharmacogenomics, an emerging field studying how genomic alterations and transcriptomic programming determine drug response, represents a potential solution [3, 4]. For instance, recent reports identified mutation profiles associated with drug response both in tumor type-specific and pan-cancer manners [5, 6]. As drug response data of large patient cohorts are scarcely available, large-scale cell line-based screening can greatly facilitate the study of pharmacogenomics in cancer. Recently, the Genomics of Drug Sensitivity in Cancer (GDSC) Project proposed a comprehensively landscape of drug response of ~ 1000 human cancer cell lines to 265 anti-cancer drugs and unveiled crucial oncogenic aberrations related to drug sensitivity [7, 8]. Because of the fundamental differences between in vitro and in vivo biological systems, a translation of pharmacogenomics features derived from cells to the prediction of drug response of tumors is to our knowledge not yet realized.

Deep learning (DL) is the state-of-the-art machine learning technology for learning knowledge from complex data and making accurate predictions. It features the ability to learn the representation of data without the need for prior knowledge and an assumption on data distributions. The DL technology has been successfully applied to bioinformatics studies of regulatory genomics, such as predicting binding motifs [9], investigating DNA variants [10], deciphering single-cell omics [11, 12], and extraction of genomics features for survival prediction [13]. In pharmaceutical and pharmacogenomics research, reports have shown its ability to predict drug-target interactions [14], screen for novel anti-cancer drugs [15], and predict drug synergy [16]. Nevertheless, data complexity and the requirement of large training datasets have limited its application to integrate genomics data and comprehensively predict drug response, hindering the translation to precision oncology.

Addressing the unmet demands, the present study is aimed to predict the response of tumors to anti-cancer drugs based on genomic profiles. We designed DeepDR, a deep neural network (DNN) model to learn the genetic background from high-dimensional mutation and expression profiles using the huge collection of tumors of The Cancer Genome Atlas (TCGA). The model was further trained by the pharmacogenomics data developed in human cancer cell lines by the GDSC Project and their corresponding genomic and transcriptomic alterations, and finally applied to TCGA data again to predict drug response of tumors. Collectively, DeepDR is a novel DL model that translates cell line-derived pharmacogenomics knowledge via tumor genomic and transcriptomic abstraction to predict tumors’ response to compound treatment.

## Methods

### Datasets

We downloaded gene-level expression data of 935 cell lines of the Cancer Cell Line Encyclopedia (CCLE) and 11,078 TCGA pan-cancer tumors from the CTD^2^ Data Portal [17] and UCSC TumorMap [18], respectively. Given the total numbers of cell lines, tumors, and genes as *C*, *T*, *G*, respectively, we metricized the expression data by ${\boldsymbol{E}}^{\boldsymbol{CCLE}}=\left\{{\mathit{\log}}_2\left({tpm}_{g,c}^{CCLE}+1\right)\right\}$, where ${tpm}_{g,c}^{CCLE}$ is the number of transcripts per million of gene *g* (*g* ∈ [1, *G*]) in cell line *c* (*c* ∈ [1, *C*]), and ${\boldsymbol{E}}^{\boldsymbol{TCGA}}=\left\{{\mathit{\log}}_2\left({tpm}_{g,t}^{TCGA}+1\right)\right\}$, where ${tpm}_{g,t}^{TCGA}$ denotes the number of transcripts per million of the same gene in tumor *t* (*t* ∈ [1, *T*]). Genes with low information burden (mean < 1 or st. dev. < 0.5) among TCGA samples were removed. Mutation Annotation Format (MAF) files of mutation data were downloaded directly from CCLE (1463 cells) [19, 20] and TCGA databases (10,166 tumors). Here we only considered four types of nonsynonymous mutations, including missense and nonsense mutations, and frameshift insertions and deletions. Thus, we had binary matrices of ${\boldsymbol{M}}^{\boldsymbol{CCLE}}=\left\{{m}_{g,c}^{CCLE}\right\}$ and ${\boldsymbol{M}}^{\boldsymbol{TCGA}}=\left\{{m}_{g,t}^{TCGA}\right\}$, where ${m}_{g,c}^{CCLE}$ and ${m}_{g,t}^{TCGA}$ are the mutation states (1 for mutation and 0 for wildtype) of gene *g* in *c* and *t*, respectively. Genes with no mutations in CCLE and TCGA samples were eliminated.

We also downloaded drug response data of 990 CCLE cell lines to 265 anti-cancer drugs measured by the half maximal inhibitory concentration (IC_50_) from the GDSC Project [7]. IC_50_ were measured in μM and represented in log scale (i.e., ${\boldsymbol{IC}}^{\boldsymbol{CCLE}}=\left\{{\mathit{\log}}_{10}\left({ic}_{d,c}^{CCLE}\right)\right\}$, with *d* denoting the *d*-th drug and *d* ∈ [1, *D*]) and missing data were imputed by a weighted mean of IC_50_ of 5 nearest drugs using R packages VIM and laeken [21, 22]. In this study, we analyzed 622 cell lines with available expression, mutation, and IC_50_ data and 9059 tumors with expression and mutation profiles.

### General settings of DNNs and computation environment

DNN training in this study were performed using the python library Keras 1.2.2 with TensorFlow backend. We used fully (or densely) connected layers for all networks. At a neuron *j*, its output *y*_*j*_ is calculated by

1 $$ {y}_j=F\left({\sum}_i{w}_{ij}{x}_i+{b}_j\right) $$

, where *x*_*i*_ is the output of neuron *i* at the previous layer of *j*, *w*_*ij*_ and *b*_*j*_ denote the synaptic weight and bias, respectively, and *F* represents an activation function. The notation of all neurons at a layer can thus be written as

2 $$ \boldsymbol{y}=F\left(\boldsymbol{wx}+\boldsymbol{b}\right). $$

During training, synaptic weights and biases are adjusted to minimize a loss function. We hereafter refer to the two parameters as synaptic parameters because they represent the model and can be used to transfer a learned model to another. In this study, DNNs were optimized using the Adam optimizer with a loss function of mean squared error (MSE). We used the He’s uniform distribution [23] to initialize autoencoders and the Prediction (P) network, while the mutation encoder (M_enc_) and expression encoder (E_enc_) in the complete model were initialized by the synaptic parameters learned from the pre-training on TCGA data. Neuron activation function was set as rectified linear unit (ReLU) except for the output layer of P as linear in order to better fit the distribution of log-scale IC_50_.

### Overview of DeepDR

**Fig. 1:**
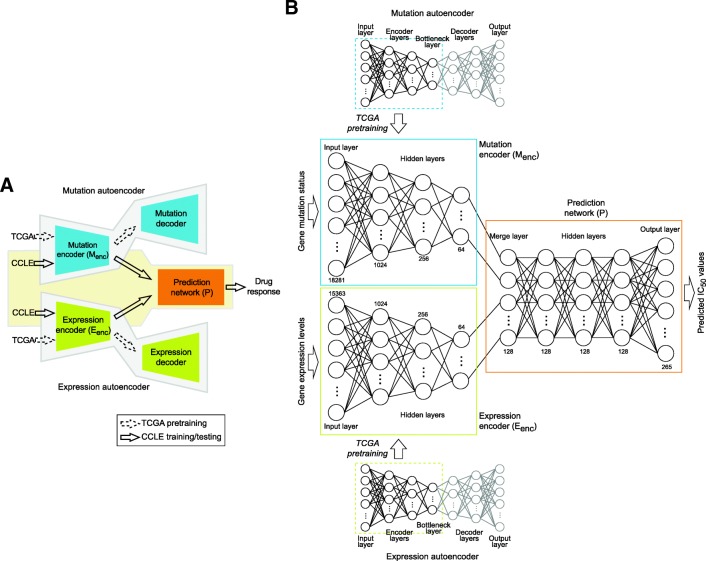
Illustration of DeepDR. (**a**) Model overview. Mutation and expression data of TCGA (*n* = 9059) were used to pre-train two autoencoders (highlighted in blue and green) to extract data representations. Encoders of the autoencoders, namely mutation encoder M_enc_ and expression encoder E_enc_, were linked to a prediction network (P; denoted in orange) and the entire network (i.e., M_enc_, E_enc_, and P) was trained using CCLE data (*n* = 622, of which 80, 10, and 10% used as training, validation, and testing, respectively) to predict the response to 265 drugs. (**b**) Architecture of the neural networks. Numbers denote the number of neurons at each layer

DeepDR was developed to predict IC_50_ values based on genomic profiles of a cell or a tumor. Given the pair of mutation and expression vectors of sample *c*, {***M***^***CCLE***^(:, *c*), ***E***^***CCLE***^(:, *c*)}, the model predicts a *D*-length vector of IC_50_, $\widehat{{\boldsymbol{IC}}^{\boldsymbol{CCLE}}}(c)$, as an output. As shown in Fig. 1, the model is composed of three networks: i) a mutation encoder (M_enc_), ii) an expression encoder (E_enc_), and iii) a prediction feedforward network (P). The first and second components are the encoding parts of two autoencoders pre-trained using TCGA data to transform high-order features of mutation and expression data into a lower dimensional representation. The encoded representations of mutation and expression profiles were linked into P and the entire model was trained on CCLE data to make prediction of IC_50_ values. Details of DeepDR are described below.

### Pre-training of mutation and expression encoders

Autoencoder is an unsupervised DL architecture that includes a symmetric pair of encoder and decoder. By minimizing the loss between input and reconstructed (i.e., decoded) data, it reduces the dimension of complex data and captures crucial features at the bottleneck layer (the layer between encoder and decoder) (Fig. 1b, top and bottom panels). We pre-trained an autoencoder on each of the TCGA mutation and expression datasets to optimize the capability to capture high-order features. To determine the optimized architecture, we adopted a hyper-parameter optimization method, namely hyperas [24], to select i) number of neurons at the 1st layer (4096, 2048, or 1024), ii) number of neurons at the 2nd layer (512, 256, or 128), iii) number of neurons at the 3rd layer (the bottleneck layer; 64, 32, or 16), and iv) batch size (128 or 64). Each combination was trained for 20 epochs; the best-performing model was re-run for 100 epochs and the synaptic parameters were saved.

### Complete prediction network

In our complete model, encoders of the two optimized autoencoders, i.e., M_enc_ and E_enc_, were linked to P to make predictions of IC_50_ (Fig. 1). P is a 5-layer feedforward neural network, including the first layer merging output neurons of the two encoders, three fully connected layers, and the last layer of *D* neurons generating IC_50_ values of *D* drugs (Fig. 1b, orange box). In the complete model, architecture (number of layers and number of neurons at each layer) of M_enc_ and E_enc_ was fixed; their synaptic parameters were initialized using the parameters obtained from pre-training in TCGA and updated during the training process. P was randomly initialized. We trained the entire model using CCLE data, with 80, 10, and 10% of samples as training, validation, and testing sets, respectively. We note the validation dataset was not used to update model parameters but to stop the training process when the loss in validation set had stopped decreasing for 3 consecutive epochs to avoid model overfitting. Performance of the model was evaluated using the testing samples, i.e., $MSE\left(\widehat{{\boldsymbol{IC}}^{\boldsymbol{C}\boldsymbol{CLE}}}\left(:,{\boldsymbol{C}}_{\boldsymbol{test}}\right),{\boldsymbol{IC}}^{\boldsymbol{C}\boldsymbol{CLE}}\left(:,{\boldsymbol{C}}_{\boldsymbol{test}}\right)\right)$, where ***C***_***test***_ denotes the test set of cell lines.

We applied the final model to predict drug response of TCGA tumors. For a tumor *t*, {***M***^***TCGA***^(:, *t*), ***E***^***TCGA***^(:, *t*)} was fed into the model and $\widehat{{\boldsymbol{IC}}^{\boldsymbol{TCGA}}}\left(:,t\right)$ was calculated. A high predicted IC_50_ indicates an adverse response of a patient to the corresponding drug.

### Comparison to other model designs

Performance of DeepDR was compared to four different DNN designs. First, to assess the effect of TCGA pre-training on M_enc_ and E_enc_, we randomly initialized both encoders using the He’s uniform distribution and calculated MSE of the entire model. Second, dimension reduction of the M_enc_ and E_enc_ networks was replaced by principal component analysis (PCA). Last two models were built without M_enc_ or E_enc_ to study whether they jointly improved the performance. In each iteration, CCLE samples were randomly assigned to training (80%), validation (10%), and testing (10%) and each model was trained and tested. Performance in terms of the number of consumed epochs and MSE in IC_50_ were summarized and compared across the 100 iterations. We also analyzed two classical prediction methods, multivariate linear regression and regularized support vector machine (SVM). For each method, top 64 principal components of mutations and gene expression were merged to predict IC_50_ values of all (using linear regression) or individual drugs (SVM).

## Results

### Construction and evaluation of DeepDR in CCLE

**Fig. 2:**
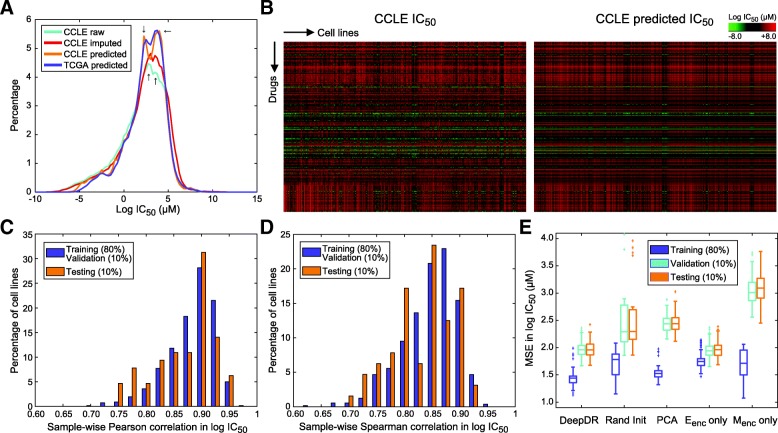
Model construction and evaluation using CCLE datasets. (**a**) Density plots of true (with missing values), imputed, and predicted IC_50_ data of CCLE and predicted data of TCGA. (**b**) Heatmaps of imputed and predicted IC_50_ data of CCLE. (**c**, **d**) Sample-wise Pearson and Spearman correlation between imputed and predicted IC_50_ data of CCLE samples. (**e**) Mean square errors of DeepDR and 4 other DNN-based designs. DeepDR was compared to a model with no TCGA pre-training (with encoders randomly initialized; abbreviated as Rand Init), with encoders substituted by PCAs, with E_enc_ only (no M_enc_), and with M_enc_ only (no E_enc_). Each model was trained for 100 times, each of which CCLE samples were randomly assigned into training, validation, and testing sets

The study is aimed to predict drug response (measured as log-scale IC_50_ values) using genome-wide mutation and expression profiles. We included mutation and expression profiles of 622 CCLE cell lines of 25 tissue types and 9059 TCGA tumors of 33 cancer types. After data preprocessing, 18,281 and 15,363 genes with mutation and expression data, respectively, available in both CCLE and TCGA samples were analyzed. Log-scale IC_50_ values of all cell lines in response to 265 anti-cancer drugs were collected from the GDSC Project [7]. After imputation of missing values, the range of log IC_50_ was from − 9.8 to 12.8 with a standard deviation of 2.6 (Fig. 2a). We designed DeepDR with three building blocks: 4-layer M_enc_ and 4-layer E_enc_ for capturing high-order features and reducing dimensions of mutation and expression data, and a 5-layer prediction network P integrating the mutational and transcriptomic features to predict IC_50_ of multiple drugs (Fig. 1). To make the best use of the large collection of TCGA pan-cancer data, we pre-trained an autoencoder for each data type and extracted the encoders, M_enc_ (number of neurons at each layer, 18,281, 1024, 256, and 64) and E_enc_ (15,363, 1024, 256, and 64), to construct our final model (detailed in Methods). Output neurons of the two encoders were linked to P (number of neurons at each layer, 64 + 64, 128, 128, 128, and 265), of which the last layer outputs predicted IC_50_. Architecture of the complete neural networks is shown in Fig. 1b.

After pre-training M_enc_ and E_enc_ components, we trained the entire model using 80% of CCLE samples together with a validation set of 10% of samples to avoid overfitting. The remaining samples (64 cells; 16,960 cell-drug combinations) were used for testing. The model achieved an overall MSE in IC_50_ of 1.53, corresponding to 1.48 and 1.98 in training/validation and testing data, respectively. Generally, the distribution of predicted IC_50_ was similar to original data (Fig. 2a-b), while the two modes of original data seemed to be enhanced (highlighted in Fig. 2a). In both training/validation and testing data, the prediction was highly consistent to the true data in terms of IC_50_ values (Pearson correlation; *ρ*_*P*_) and rank of drugs (Spearman correlation; *ρ*_*S*_) of a sample (*ρ*_*P*_ ∈ [0.70, 0.96], *ρ*_*S*_ ∈ [0.62, 0.95], and all *P*-values < 1.0 × 10^− 29^; Fig. 2c-d). Of note, correlations achieved in training/validation and testing samples were highly comparable (Fig. 2c-d), confirming the performance of our model.

### Performance comparisons to other designs

To test the stability of DeepDR, we ran 100 training processes each of which training, validation, and testing cells were reselected. Overall, the model converged in 14.0 epochs (st. dev., 3.5; Table 1) and achieved an MSE of 1.96 in testing samples (st. dev., 0.13; Fig. 2e and Table 1). We compared the performance to linear regression, SVM, and four analog DNNs of DeepDR, including random initialization (identical architecture, but without TCGA pre-training of M_enc_ and E_enc_), PCA (M_enc_ and E_enc_ each replaced by top 64 principal components of mutation and expression data), M_enc_ only (E_enc_ removed from DeepDR), and E_enc_ only (M_enc_ removed from DeepDR). The two classical methods seemed to suffer from high MSE in testing samples (10.24 and 8.92 for linear regression and SVM, respectively; Table 1). DeepDR also outperformed DNNs with random initialization and PCA in MSE (difference in medians, 0.34 and 0.48; Fig. 2e and Table 1) and stability (st. dev. of MSE in testing samples = 0.13, 1.21, and 0.17 for DeepDR, random initialization, and PCA, respectively; Fig. 2e). While the E_enc_-only model achieved similar performance to our model (difference in medians = 0.0042; Fig. 2E and Table 1), the addition of M_enc_ seemed to bring faster convergence (difference in medians = 3; Table 1). Our data echoed the biological premise that gene expressions are more directly linked to biological functions and thus richer in information burden than mutations.

**Table 1:** Performance of DeepDR and other models

Measurement	DeepDR	Linear regression	SVM	Random initialization	PCA	E_enc_ only	M_enc_ only
Median MSE in testing samples^a^	1.96	10.24^b^	8.92^c^	2.30	2.44	1.96	3.09
Median number of training epochs^a^	14	–	–	9	29	17	9.5

^a^Median of 100 shuffles of training, validation, and testing samples

^b^Result of one multivariate regression model

^c^Results of 265 SVM models, each predicting IC_50_ for a drug

### Associations of gene mutations to predicted drug response in TCGA – Per-cancer study

**Fig. 3:**
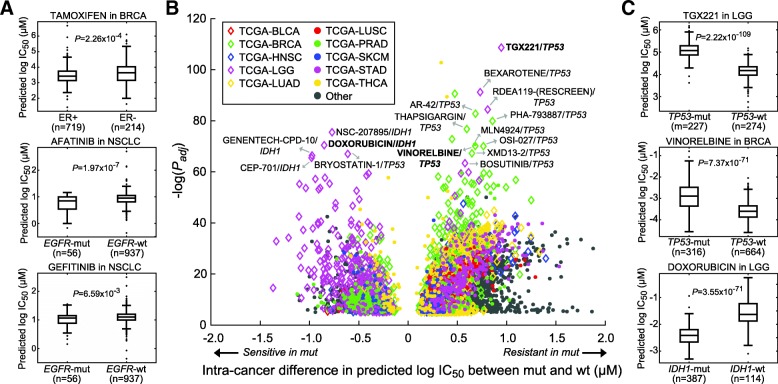
Associations of gene mutations to predicted drug response in TCGA – per-cancer study. (**a**) Predicted IC_50_ of TCGA tumors with known drug targets in a cancer type. Significance of ΔIC_50_ between tumors with and without a gene mutation was assessed by the two-tailed *t*-test. (**b**) Significant mutation–drug pairs in each cancer with Bonferroni adjusted *t*-test *P* < 1.0 × 10^−5^. Nodes labeled with names are those with extreme significance (adjust *P* < 1.0 × 10^−60^) and magnitude of ΔIC_50_ (|ΔIC_50_| ≥ 0.5). Top 10 cancer types with the largest sample sizes are denoted by node color and shape. (**c**) Box plots of three mutation–drug examples in BRCA and LGG

In search of effective anti-cancer drugs in tumors, we applied DeepDR directly to predict the response of 9059 TCGA samples to the 265 anti-cancer drugs. The predicted IC_50_ values followed a similar distribution to CCLE cells (Fig. 2a, blue line). Realizing the different nature of cell lines and tumors, we started by examining several drugs with well-known target genes. As shown in Fig. 3a, breast invasive carcinoma (BRCA) with positive estrogen receptor (ER; assessed by immunohistochemistry by TCGA) responded to a selective estrogen receptor modulator, tamoxifen, significantly better than ER-negative patients (*t*-test *P* = 2.3 × 10^− 4^). Also, two EGFR inhibitors, afatinib and gefitinib, achieved better performance in non-small cell lung cancers (NSCLC) with mutated *EGFR* (*P* = 2.0 × 10^− 7^ and 6.6 × 10^− 3^). While the promising results on these well-characterized drugs showed the applicability of our model to tumors, we noted that the magnitude of differences in predicted IC_50_ levels was modest, underlining the fundamental differences between cell lines and tumors. In order to prioritize mutations underlying drug response, we systematically analyzed all cancer–mutation–drug combinations and tested the significance of differences in IC_50_ between samples with and without a mutation for each cancer. Here only genes with a mutation rate higher than 10% and harbored by at least 10 patients in a cancer were analyzed. With a stringent criterion of Bonferroni-adjusted *t*-test *P* < 1.0 × 10^− 5^, we identified a total of 4453 significant cancer–mutation–drug combinations involving 256 drugs and 169 cancer–mutation combinations (Fig. 3b). The top three combinations were *TP53* mutations in lung adenocarcinoma (LUAD; modulating response to 235 drugs), lung squamous cell carcinoma (LUSC; 228 drugs), and stomach adenocarcinoma (STAD; 224 drugs) (Table 2). *TP53* was one of the most frequently mutated and well-studied genes in many cancers. The mutation has been shown to be associated with cancer stem cells and resistance functions and thus regulates drug resistance [25, 26]. For instance, our data indicated its associations with resistance of a PI3Kβ inhibitor, TGX221, in 9 cancers including low-grade glioma (LGG; mean difference in IC_50_ (ΔIC_50_) = 0.95; *P* = 2.2 × 10^− 109^; Fig. 3c) and resistance of vinorelbine in BRCA (ΔIC_50_ = 0.68; *P* = 7.4 × 10^− 71^; Fig. 3c) and 6 other cancers. We also identified gene mutations that sensitized tumors to a large number of drugs, such as *IDH1* (138 drugs; Table 2). *IDH1* was the most frequently mutated gene in LGG (77.3% in our data; Table 2) and known to regulate cell cycle of glioma cells and enhance the response to chemotherapy [27]. Our finding agreed with the report and showed that *IDH1* mutation dramatically reduced IC_50_ of chemotherapeutic agents, e.g., doxorubicin in LGG (ΔIC_50_ = − 0.85; *P* = 3.6 × 10^− 71^; Fig. 3c).

**Table 2:** Top mutations in modulating drug response among individual cancers

Cancer	Gene	Mutation rate	Num. modulated drugs	Num. sensitive drugs	Num. resistant drugs
LUAD	*TP53*	46.1%	235	0	235
LUSC	*TP53*	75.1%	228	0	228
STAD	*TP53*	43.3%	224	0	224
HNSC	*TP53*	66.1%	207	0	207
COAD	*TP53*	55.7%	197	0	197
LIHC	*TP53*	27.0%	194	1	193
BRCA	*TP53*	32.2%	182	7	175
LGG	*IDH1*	77.3%	159	138	21
PRAD	*TP53*	10.8%	146	1	145
KIRC	*PBRM1*	38.0%	142	3	139

### Associations of gene mutations to predicted drug response in TCGA – Pan-cancer study

**Fig. 4:**
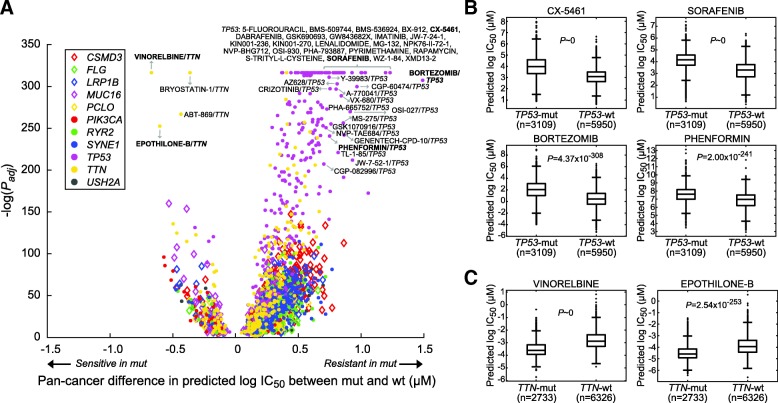
Associations of gene mutations to predicted drug response in TCGA – pan-cancer study. (**a**) Gene mutations significantly associated with predicted drug response across all TCGA samples. Here only the 11 genes with mutation rates larger than 10% were analyzed. Nodes labeled with names are those with extreme significance (adjust *P* < 1.0 × 10^−200^) and magnitude of ΔIC_50_ (ΔIC_50_ ≥ 0.7 or ΔIC_50_ < 0). (**b**, **c**) Examples of drugs modulated by *TP53* and *TTN* mutations, respectively

We also carried out a study to explore how gene mutations affect drug response in a pan-cancer setting. The analysis was focused on 11 genes with mutation rates higher than 10% across all TCGA samples (Table 3). Using an identical criterion, we identified 2119 significant mutation–drug pairs composed of 256 drugs, among which 1882 (88.8%) and 237 (11.2%) were more resistant and sensitive in mutated samples, respectively (Fig. 4a and Table 3). *TP53* (251 drugs), *CSMD3* (223), *SYNE1* (218), *TTN* (206), and *RYR2* (199) were the top drug response-modulating genes (Table 3). Among them, *TP53* (9 sensitive and 242 resistant drugs) and *TTN* mutations (44 and 162) were associated with the largest numbers of resistant and sensitive drugs, respectively (Table 3). Thus, we further investigated drugs associated with the 2 genes. Many of the drugs with large *TP53* mutations-modulated changes in ΔIC_50_ (|ΔIC_50_| ≥ 0.7; Fig. 4a-b) were previously studied in different cancer types by in vitro models. For instance, wildtype *TP53* is required in the anti-cancer actions of CX-5461 [28, 29] and sorafenib [30] (both *P* of ΔIC_50_ ~ 0 in our data; Fig. 4b), sensitizes various cancer cells to bortezomib [31] (*P* = 4.4 × 10^− 308^; Fig. 4b), and enhances phenformin-induced growth inhibition and apoptosis [32] (*P* = 2.0 × 10^− 241^; Fig. 4b). As for previously less explored *TTN* mutations, the longest gene in human genome known to carry a large number of variations, our data indicated that perhaps *TTN* acts as a marker gene of tumors sensitized to chemotherapeutic agents such as vinorelbine (*P* ~ 0; Fig. 4C) and a potential anti-cancer drug epothilone B (*P* = 2.5 × 10^− 253^; Fig. 4c). Taken together findings from our per- and pan-cancer studies, we have demonstrated the applicability of our model to predict drug response of tumors and unveil novel and well-studied genes modulating drug response in cancer.

**Table 3:** Top gene mutations modulating pan-cancer drug response

Gene	Mutation rate	Num. modulated drugs	Num. sensitive drugs	Num. resistant drugs
*TP53*	34.3%	251	9	242
*CSMD3*	12.6%	223	12	211
*SYNE1*	11.5%	218	10	208
*TTN*	30.2%	206	44	162
*RYR2*	11.9%	199	14	185
*USH2A*	10.7%	191	12	179
*LRP1B*	12.1%	188	19	169
*FLG*	11.0%	183	9	174
*MUC16*	19.5%	161	51	110
*PCLO*	10.5%	155	12	143
*PIK3CA*	11.7%	144	45	99

### Pharmacogenomics analysis of docetaxel and CX-5461 in TCGA

**Fig. 5:**
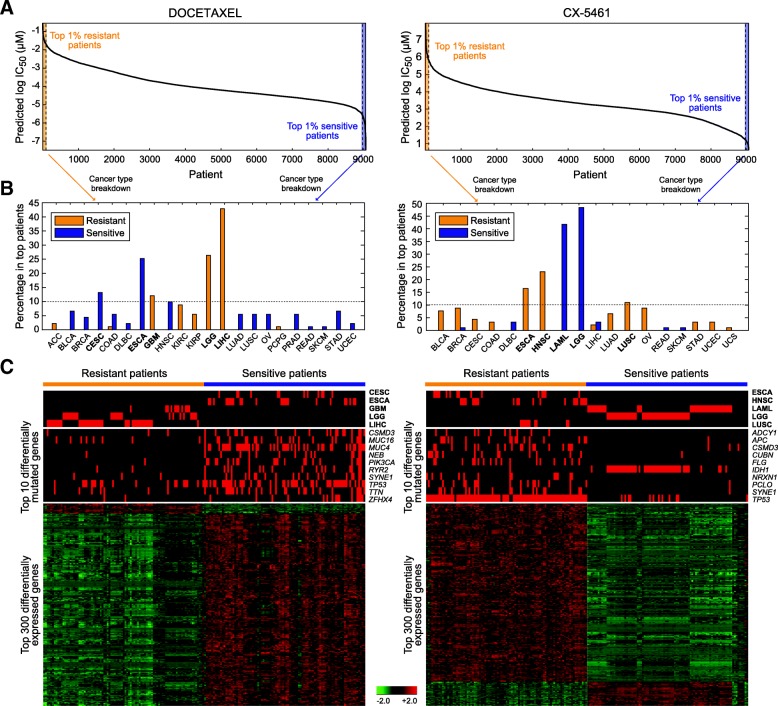
Pharmacogenomics analysis of docetaxel and CX-5461 in TCGA. (**a**) Waterfall plot of predicted IC_50_ for the two drugs across all TCGA samples. Tumors with extreme IC_50_ values (top and bottom 1%) were denoted as the resistant and sensitive groups. (**b**) Cancer type composition of resistant and sensitive samples. Cancer types accounted for at least 10% in any group are highlighted in bold and shown in (**c**). (**c**) Heatmaps of cancer type composition, top differentially mutated genes, and top differentially expressed genes between the two groups. In the expression heatmap, genes are normalized and hierarchically clustered, and samples are clustered within each group

To unveil the pharmacogenomics landscape of drugs, a comprehensive study of mutation and expression profiles associated with resistance of a drug in a pan-cancer setting was carried out. Here we took two drugs as demonstrating examples, a widely used chemotherapeutic agent docetaxel and a novel anti-cancer drug CX-5461 currently under investigation in several cancers. For each drug, pan-cancer patients predicted to be very sensitive and resistant (with IC_50_ in bottom and top 1%, *n* = 91 in each group; Fig. 5a, left panel) were compared for cancer type compositions, mutation rates, and differential gene expression. Top cancer types of docetaxel-sensitive patients were esophageal carcinoma (ESCA; 25.3%), cervical and endocervical cancer (CESC; 13.2%), and head and neck squamous cell carcinoma (HNSC; 9.9%) (Fig. 5b, left panel), while top resistant patients were mainly liver hepatocellular carcinoma (LIHC; 42.9%), LGG (26.4%), and glioblastoma multiforme (GBM; 12.1%) (Fig. 5b, left panel). Top 10 gene with most changed mutation rates between the two groups of patients are listed in Fig. 5c. On average, each sensitive tumor harbored 2.7 mutations among these genes, much higher than 0.51 observed in the resistant group (Fig. 5c, left panel), implying tumors with higher mutation burdens in crucial genes may be more vulnerable to the treatment. Of note, a great majority of the most significantly differentially expressed genes were upregulated in sensitive patients (Fig. 5c, left panel). We performed functional annotation analysis of the top 300 genes in Gene Ontology terms of biological processes and molecular functions using the Database for Annotation, Visualization and Integrated Discovery (DAVID) v6.7 [33, 34]. While we did not observe any cluster of functions related to microtubule, through which docetaxel physically binds to the cell and regulate the cell cycle [35], these drug sensitivity-related genes were indeed predominantly enriched in functions governing the mitotic cell cycle (Table 4). The observation largely reflected the nature of the chemotherapeutic agent to target highly proliferative cells and the dependence of drug response on the ability to pass cell-cycle checkpoints. In addition to docetaxel, we analyzed a novel anti-cancer agent, CX-5461. This inhibitor of ribosomal RNA synthesis has been shown with anti-cancer properties in cancer cells [36, 37] and is now under phase I/II clinical trial in solid tumors (NCT number, NCT02719977). In hematopoietic malignancies, it was recently shown to outperform standard chemotherapy regimen in treating aggressive acute myeloid leukemia (LAML) [29], and its anti-cancer effects were dependent on wild-type *TP53* [28, 29]. Concordantly, in our data, LAML and lymphoid neoplasm diffuse large B-cell lymphoma (DLBC) jointly accounted for 45.1% (41.8 and 3.3%) of patients predicted be respond extremely well to CX-5461 (Fig. 5a-b, right panels). Of note, LGG comprised another 48.4% of the sensitive tumors (Fig. 5b, right panel). Nine of the top 10 differentially mutated genes were enriched in the resistant group and led by *TP53* mutations (mutation rate, 95.6% in resistant vs. 13.2% in sensitive patients; Fig. 5c, right panel), echoing data from our pan-cancer analysis (Fig. 4a-b) and previous in vitro and in vivo investigations [28, 29]. *IDH1* was the only gene preferentially mutated in sensitive tumors and largely marked LGG (mutated in 42 of 44 sensitive LGG; Fig. 5C, right panel). DAVID analysis of the top 300 differentially expressed genes highlighted differential mechanisms between solid and non-solid tumors, such as extracellular matrix and cell motion (Table 5). Altogether, the pharmacogenomics analyses revealed well-known resistance mechanisms of docetaxel and shed light on the potential of CX-5461 on hematopoietic malignancies and LGG.

**Table 4:** Top GO clusters enriched in top 300 differentially expressed genes associated with predicted response to docetaxel

GO ID	GO term	Num. genes	*P*-value
Cluster 1 (enrichment score: 10.89)			
GO:0007049	cell cycle	40	1.13 × 10^−10^
GO:0022402	cell cycle process	33	3.51 × 10^− 10^
GO:0000279	M phase	32	1.01 × 10^−15^
Cluster 2 (enrichment score: 3.96)			
GO:0000166	nucleotide binding	56	1.95 × 10^−4^
GO:0032553	ribonucleotide binding	54	2.74 × 10^−6^
GO:0032555	purine ribonucleotide binding	54	2.74 × 10^−6^
Cluster 3 (enrichment score: 3.45)			
GO:0000278	mitotic cell cycle	26	1.01 × 10^− 9^
GO:0051726	regulation of cell cycle	15	8.48 × 10^−4^
GO:0007346	regulation of mitotic cell cycle	12	3.09 × 10^−5^
Cluster 4 (enrichment score: 2.47)			
GO:0051327	M phase of meiotic cell cycle	8	9.46 × 10^−4^
GO:0007126	meiosis	8	9.46 × 10^−4^
GO:0051321	meiotic cell cycle	8	1.07 × 10^− 3^
Cluster 5 (enrichment score: 2.07)			
GO:0051276	chromosome organization	13	8.64 × 10^−2^
GO:0007059	chromosome segregation	6	9.34 × 10^− 3^
GO:0000070	mitotic sister chromatid segregation	5	2.45 × 10^− 3^

Each cluster is represented by the largest three GO terms

**Table 5:** Top GO clusters enriched in top 300 differentially expressed genes associated with predicted response to CX-5461

GO ID	GO term	Num. genes	*P*-value
Cluster 1 (enrichment score: 8.65)			
GO:0043062	extracellular structure organization	17	2.93 × 10^−9^
GO:0030198	extracellular matrix organization	15	4.55 × 10^−10^
GO:0005201	extracellular matrix structural constituent	13	2.64 × 10^−9^
Cluster 2 (enrichment score: 6.13)			
GO:0008544	epidermis development	18	2.35 × 10^−9^
GO:0007398	ectoderm development	18	7.71 × 10^−9^
GO:0030855	epithelial cell differentiation	8	4.60 × 10^−3^
Cluster 3 (enrichment score: 4.23)			
GO:0030199	collagen fibril organization	9	7.34 × 10^−9^
GO:0032963	collagen metabolic process	6	5.37 × 10^−5^
GO:0044259	multicellular organismal macromolecule metabolic process	6	8.96 × 10^−5^
Cluster 4 (enrichment score: 2.84)			
GO:0006928	cell motion	18	8.22 × 10^−4^
GO:0016477	cell migration	13	9.51 × 10^−4^
GO:0048870	cell motility	13	2.33 × 10^−3^
Cluster 5 (enrichment score: 2.60)			
GO:0060429	epithelium development	12	6.39 × 10^−4^
GO:0030855	epithelial cell differentiation	8	4.60 × 10^−3^
GO:0009913	epidermal cell differentiation	6	4.49 × 10^−3^

Each cluster is represented by the largest three GO terms

## Discussion

DNN is unquestionably one of the largest computational breakthroughs in the era of big data. Although promising results of our and other studies have demonstrated its ability of solving challenging bioinformatic tasks, the method has several fundamental limitations. For instance, due to high representational power and model complexity, the method suffers from overfitting and the requirement of large training data. Addressing this, the present study adopts a training–validation partition of training data to allow early stopping to the training process [38]. Future work may further incorporate dropout and regularization to DNNs. Also, by taking advantage of the transferability of neural networks, we used the huge volume of TCGA data to equip our model the ability of capturing representations of mutation and expression data. Transferring the learned parameters to initialize our model virtually increased the sample size of our training data. Our data from 100 iterations of model training suggest the stability of performance and insensitivity to the selection of training samples. With the availability of more large-scale drug screening data, we expect the proposed model to make even more accurate predictions and unveil subtle pharmacogenomics features. Furthermore, DeepDR may incorporate additional genomic mutation information, such as copy number alterations, into data matrices ***M***^***TCGA***^ and ***M***^***CCLE***^, to enrich the complexity of tumor mutation for model training and further reduce the training MSE. Because of the nature of DNNs as black boxes, the interpretability of results is typically limited. In this study, by integrating genomics profiles to the predictions, we systematically investigated how single gene mutations, as well as the interplay between cancer type, mutations, and biological functions, were associated with the predicted drug response. Several novel methods were recently proposed to extract features learned by neural networks, such as network-centric approach [39] and decomposition of predicted outputs by backpropagation onto specific input features [40] (reviewed in [41]). Future works may incorporate these methods to provide a landscape of pharmacogenomics and further reveal novel oncogenic genomics profiles.

## Conclusions

This study addresses the need for a translation of pharmacogenomics features identified from pre-clinical cell line models to predict drug response of tumors. We developed a DNN model capable of extracting representative features of mutations and gene expression, and bridging knowledge learned from cancer cell lines and applications to tumors. We showed the reliability of the model and its superior performance than four different methods. Applying our model to the TCGA collection of tumors, we identified both well-studied and novel resistance mechanisms and drug targets. Overall, the proposed model is widely applicable to incorporate other omics data and to study a wider range of drugs, paving the way to the realization of precision oncology.

## Abbreviations

ACC: adrenocortical cancer
BLCA: bladder urothelial carcinoma
BRCA: breast invasive carcinoma
CCLE: Cancer Cell Line Encyclopedia
CESC: cervical and endocervical cancer
CHOL: cholangiocarcinoma
COAD: colon adenocarcinoma
DL: deep learning
DLBC: diffuse large B-cell lymphoma
DNN: deep neural network
E_enc_: expression encoder
ER: estrogen receptor
ESCA: esophageal carcinoma
GBM: glioblastoma multiforme
HNSC: head and neck squamous cell carcinoma
IC_50_: half maximal inhibitory concentration
KICH: kidney chromophobe
KIRC: kidney clear cell carcinoma
KIRP: kidney papillary cell carcinoma
LAML: acute myeloid leukemia
LGG: lower grade glioma
LIHC: liver hepatocellular carcinoma
LUAD: lung adenocarcinoma
LUSC: lung squamous cell carcinoma
M_enc_: mutation encoder
MESO: mesothelioma
MSE: mean squared error
MUT: mutated
NSCLC: non-small cell lung cancer
Num: number
OV: ovarian serous cystadenocarcinoma
P: prediction network
*P*: *P*-value
PCA: principal component analysis
PCPG: pheochromocytoma and paraganglioma
PRAD: prostate adenocarcinoma
Rand Init: random initialization
READ: Rectum adenocarcinoma
SARC: Sarcoma
SKCM: Skin cutaneous melanoma
STAD: Stomach adenocarcinoma
SVM: Support vector machine
TCGA,: The Cancer Genome Atlas
TGCT: Testicular germ cell tumor
THCA: Thyroid carcinoma
THYM: Thymoma
UCEC: Uterine corpus endometrioid carcinoma
UCS: Uterine carcinosarcoma
UVM: Uveal melanoma
WT: Wildtype
