# Correction to: Predicting drug response of tumors from integrated genomic profiles by deep neural networks

**DOI:** 10.1186/s12920-019-0569-5

**Published:** 2019-08-12

**Authors:** Yu-Chiao Chiu, Hung-I Harry Chen, Tinghe Zhang, Songyao Zhang, Aparna Gorthi, Li-Ju Wang, Yufei Huang, Yidong Chen

**Affiliations:** 1 Greehey Children’s Cancer Research Institute, University of Texas Health Science Center at San Antonio, San Antonio, TX 78229 USA; 2 Department of Electrical and Computer Engineering, The University of Texas at San Antonio, San Antonio, TX 78249 USA; 3 Laboratory of Information Fusion Technology of Ministry of Education, School of Automation, Northwestern Polytechnical University, Xi’an, 710072 Shaanxi China; 4 Department of Epidemiology and Biostatistics, University of Texas Health Science Center at San Antonio, San Antonio, TX 78229 USA

**Correction to: BMC Medical Genomics 2019, 12(Suppl 1):18.**

**https://doi.org/10.1186/s12920-018-0460-9**

Following publication of the original article [1], the authors provided an updated funding statement to the article. The updated statement is as follows:

## Funding

This research and this article’s publication costs were supported partially by the NCI Cancer Center Shared Resources (NIH-NCI P30CA54174 to YC), NIH (CTSA 1UL1RR025767–01 to YC, and R01GM113245 to YH), CPRIT (RP160732 to YC), and San Antonio Life Science Institute (SALSI Innovation Challenge Award 2016 to YH and YC and SALSI Postdoctoral Research Fellowship to YCC). AG is supported by the NIH/NCATS TL1 Translational Science Training award (TL1TR002647) and the AACR-AstraZeneca Stimulating Therapeutic Advances through Research Training grant. The funding sources had no role in the design of the study and collection, analysis, and interpretation of data and in writing the manuscript.
